# The Effects of Log-in Behaviors and Web Reviews on Patient Consultation in Online Health Communities: Longitudinal Study

**DOI:** 10.2196/25367

**Published:** 2021-06-03

**Authors:** Qin Chen, Jiahua Jin, Tingting Zhang, Xiangbin Yan

**Affiliations:** 1 School of Economics and Management University of Science and Technology Beijing Beijing China

**Keywords:** online health communities, digital health, patient consultation, log-in behavior, web reviews, offline status

## Abstract

**Background:**

With the rapid development of information technology and web-based communities, a growing number of patients choose to consult physicians in online health communities (OHCs) for information and treatment. Although extant research has primarily discussed factors that influence the consulting choices of OHC patients, there is still a lack of research on the effects of log-in behaviors and web reviews on patient consultation.

**Objective:**

This study aims to explore the impact of physicians’ log-in behavior and web reviews on patient consultation.

**Methods:**

We conducted a longitudinal study to examine the effects of physicians’ log-in behaviors and web reviews on patient consultation by analyzing short-panel data from 911 physicians over five periods in a Chinese OHC.

**Results:**

The results showed that the physician’s log-in behavior had a positive effect on patient consultation. The maximum number of days with no log-ins for a physician should be 20. The two web signals (log-in behavior and web reviews) had no complementary relationship. Moreover, the offline signal (ie, offline status) has different moderating effects on the two web signals, positively moderating the relationship between web reviews and patient consultation.

**Conclusions:**

Our study contributes to the eHealth literature and advances the understanding of physicians’ web-based behaviors. This study also provides practical implications, showing that physicians’ log-in behavior alone can affect patient consultation rather than complementing web reviews.

## Introduction

### Background

With the development of Health 2.0 technologies, the number of people using the internet to meet their health-related needs is increasing [1]. Online health communities (OHCs) have become prevalent in recent years, and research has focused on two of them: web-based physician-patient communities and web-based patient communities. A web-based physician-patient community is a platform that connects physicians with patients where patients can consult physicians on health issues and disease treatments anytime and anywhere. The object of this study is this kind of OHC, namely, the web-based physician-patient community.

Unlike other types of services, health care services have several characteristics. First, the disease of each patient is unique [2]. Second, life and death matter [3]. Third, serious information asymmetry exists between physicians and patients [4]. The choice of an appropriate physician has always been the focus of research in the health care field. The emergence of OHCs has effectively alleviated the problem of information asymmetry between physicians and patients. Unlike traditional health care services, OHCs give patients the opportunity to review the abundant amount of information about various physicians and then use this information to choose the physician whom they want to consult [5]. Although numerous studies have explored the factors that influence patients’ choices of consulting [5-14], they are not the only ones; thus, more research is needed.

As health care service providers, physicians’ web behaviors, such as knowledge-sharing behaviors [9], interactions with patients [5,6], and written and telephone consultations [13], provide important information when patients make consulting choices. However, for physicians, the premise of providing web-based health care services and conducting these behaviors involves logging into their accounts of the OHC. As physicians work full time in hospitals or clinics [14], they can only use off-duty hours to provide web-based services [15], which suggests that there are different log-in patterns in OHCs. Web-based behaviors reflect the degree of the physician’s activeness and effort, as well as the quality of the service process [5,6,14,16]. In addition, as one kind of information generated by patients who have experienced health care services, web reviews can reflect the service outcome [14,16] and influence patients to make consulting choices [8-10]. The assessment of service quality should focus on both the outcome and delivery process they receive [17]. Log-in behavior and web reviews were evaluated in this study. Therefore, whether log-in behavior is related to patient consultation and its relationship with web reviews is worth studying.

As a web-based health platform may provide multiple signaling mechanisms simultaneously [18], there are multiple signals that influence patient consultation. This study focuses on both web-based and offline signals. According to the signaling theory [19], which indicates how the information receiver interprets signals along with information from the sender, log-in behaviors and web reviews can be considered as two web signals provided by a physician to assist patients in making consulting choices [6,11,20]. In addition, considering that people live in a mixed environment comprising web-based and offline worlds, the status of a physician’s offline world (ie, offline status) as an offline signal may affect the relationship between web signals and patient consultation.

The objective of this study is to investigate how a physician’s log-in behavior and web reviews affect patients’ choices for consultation using data from a Chinese OHC. The main research questions are as follows:

1. How does a physician’s log-in behavior affect patient consultation in OHCs?
2. How does a physician’s log-in behavior and web reviews complement each other in affecting patient consultation?
3. How does the offline status of physicians moderate the effects of web signals (ie, log-in behavior and web reviews) on patient consultation?

To answer these three research questions, we collected data from 911 physicians over five periods and proposed a research model based on the signaling theory.

### Related Research

With the development of information technologies, many physicians and patients are using OHCs. An OHC is a web-based community that presents a medical ecosystem, including patients and physicians, and is a core communication platform wherein patients can obtain physicians’ web-based services, knowledge about diseases, and emotional support [20,21]. As health care service characteristics [2-4], the patient’s choice of an appropriate physician for health care consultation has been the focus of research in the health care field. Table 1 summarizes the studies on patient consultation with OHCs. Although there are many factors that influence patients’ choices of consulting [5-14], not all of them have been studied. More research is needed to better understand how information affects patient consultation in OHCs.

**Table 1:** Studies of patient consultation in online health communities.

Study	Theory	Influencing factors
Cao et al [5]	ELM^a^	The number of current patients who repeatedly interact with the physician, voting heating, service star, disease knowledge, and disease risk
Deng et al [6]	N/A^b^	Physician effort and web reputation
Li et al [7]	ELM	Technical quality, interpersonal quality, votes, high-privacy disease, and private doctor service
Li et al [9]	N/A	Web-based rating and activeness
Li et al [8]	N/A	Technical skills, interpersonal skills, and gender
Liu et al [11]	Signaling theory	The physician’s web reputation and offline reputation; the hospital’s web reputation and offline reputation
Liu et al [10]	N/A	Web-based service reviews, offline service reviews, and disease risk
Lu and Wu [12]	Service quality theory	Technical quality, functional quality, and disease risk
Wu and Lu [13]	N/A	Written consultation, telephone consultation, and doctor reputation
Yang et al [14]	Signaling theory	System-generated information and patient-generated information

^a^ELM: elaboration likelihood model.

^b^N/A: not applicable.

An OHC is a web-based platform wherein physicians can provide more types of health care services than offline hospitals or clinics [22], such as network consultation, phone consultation, and appointment registration. Furthermore, physicians can update their personal information, publish articles, respond to consultations, and manage patients. In the literature, many scholars have investigated physicians’ web-based behaviors, such as knowledge-sharing behaviors [9,23,24], interactions with patients [5,6], written and telephone consultations [13], and contribution behaviors [25-27].

Although several web-based behaviors of physicians have been studied, not all of them have been evaluated. Log-in behavior is a web-based behavior of physicians that involves launching profile home pages in health websites and logging into their accounts. Log-in behavior is the first step for physicians to participate in OHCs and conduct other web-based behaviors. Many physicians offer services in both OHCs and hospitals or clinics. Owing to the heavy workload at hospitals or clinics (offline services), they can only use their spare time to provide patients with web-based services [15]. Thus, physicians have unique log-in behaviors. Previous research has shown that information about physicians’ web-based behaviors is an important factor influencing patients’ consultation choices [5,6,13]. However, less attention has been paid to log-in behaviors in OHCs. Therefore, the aim of this study is to explore physicians’ log-in behaviors and their roles in OHC consulting choices.

### Research Model and Hypotheses

#### Signaling Theory

The signaling theory is used to describe the behaviors of two parties (individuals or organizations) when accessing different information and has been applied in studies of investment decisions, entrepreneur-investor relationships [4], and web-based social trading [28]. The primary parties in the signaling theory include signalers and receivers as well as the signal itself. A signaler sends signals to the receiver to reflect quality [19]. The receiver evaluates the quality of the signaler and acts. As the two parties hold different amounts and levels of information, significant information asymmetry exists between the signalers and receivers [29]. Hence, the signal conveyed by signalers affects the degree of information asymmetry and can affect the receivers’ behaviors.

Parties in OHCs include physicians and patients. Patients are at a disadvantage, as they must rely on physicians to provide health care services [30]. Physicians, as signalers, can provide information (eg, titles, workplaces, web-based behaviors, or reviews) to receivers (patients) [11], which can help patients choose physicians to serve their needs. Referring to past studies [11,14], this study used the signaling theory to explain the effects of log-in behavior and web reviews on patient consultation choices.

#### Log-in Behavior and Web Reviews

For patients, web-based behavior is often an important factor in choosing a physician. On the one hand, web-based behavior indicates the level of active participation that stems from internet motivation within the web-based community. Activeness has a certain influence on the number of patient consultations [9]. On the other hand, web-based behavior is a positive indicator of a physician’s effort and popularity. Patients can gain insight into a physician’s past efforts through web-based behaviors, which may influence their attitudes toward the physician, thereby influencing the likelihood of selecting that physician [6]. Most importantly, physicians’ web-based behaviors are important cues for evaluating service process quality [14,16]. Combined with health care service characteristics [2-4], patients prefer to choose physicians who can provide a high-quality service process.

As the web-based behavior of a physician, log-in behavior reflects the degree of active participation in the OHC and the physician’s efforts. Given that a physician may log in many times each day to check for new messages, log-in is the basis for any active actions for physicians in OHCs. Li et al [31] believed that log-in behavior belongs to the central working sphere, and log-in patterns could indicate a physician’s central efforts related to the work, as well as the physician’s attitude toward service provision, participation degree, and responsibility. Bitner et al [32] revealed that customers’ perceptions of service depend on service providers’ efforts, and their behaviors will raise the purchase intentions or continuous purchase intentions of customers [33], thus having a positive effect on marketing sales or organizational performance [34]. Physicians as providers of web-based health care services also apply to this phenomenon [9]. On the one hand, physicians with a higher-frequency log-in are more likely to make more task-related efforts to attract a greater number of patients, and subsequent patients would consider this for references. On the other hand, a higher-frequency log-in appears to be more responsive and involved than others [35], with those physicians logging in more frequently being more likely to ensure timeliness in service delivery, leading to attracting more patients. Therefore, the following hypothesis is proposed:

Hypothesis 1: a physician’s log-in behavior has a positive effect on patient consultation.

Web reviews are a particular type of user-generated content or electronic word-of-mouth, are the most important information source in customers’ decision-making processes [36], and are deemed more successful in influencing customer behaviors than traditional marketing, information provided by products or service providers, or promotion messages from third-party websites [37-39].

OHCs provide a feedback channel where patients can express their views on the physician’s service and share treatment experiences on the web. This information can help patients understand a physician’s service quality at a minimal cost. In OHCs, web reviews are generated by patients who have experienced health care services. The more web reviews about a physician presented in the OHC, the more patients have selected the physician for consultation [16]. The web reviews generated by patients with similar experiences are more objective and credible signals than traditional information from acquaintances [40], which can increase other patients’ trust in the physician and reduce perceived risks [41]. Web reviews are signals that reflect a physician’s service outcome [14,16]. Positive web reviews mean a higher outcome quality of the physician, which has been shown to influence patients to make consulting choices [5,8,9,12,14].

The coexistence of log-in behavior and web reviews may complement each other in driving patient consultation. As service is delivered via the interaction between the service provider and the receiver, the assessment of service quality includes not only the delivery process but also the outcome [17]. As per the preceding discussion, log-in behavior may send web signals reflecting the service delivery process from the physicians themselves. A physician with a positive log-in behavior is usually associated with a positive attitude toward the consultation service. Furthermore, web reviews send another web signal from patients who have visited the physician before, which represents the service outcome. A physician with positive web reviews is usually associated with positive outcome quality. On the basis of the characteristics of health care services [2-4], patients judging a physician rely on two types of web signals: service process quality (ie, log-in behavior) and service outcome quality (ie, web reviews). Physicians with both high outcome quality and process quality are scarce resources [18], and the demand of these physicians on the platform should be large, so a large number of patients choose these patients. As a result, log-in behavior and web reviews should complement each other. From the preceding discussion, we propose the following hypothesis:

Hypothesis 2: a physician’s log-in behavior and web reviews have a complementary relationship that affects patient consultation.

#### Moderating Effect of Offline Status

Offline status reflects a physician’s abilities and performance in providing health care services in hospitals or clinics [11,13], referring to a physician’s career titles, ranking, and position in the hospital [11,42]. Such information can help patients evaluate a physician’s offline competence [9].

In traditional health care services, patients can only judge a physician’s ability through limited information. In the case of other factors being considered to be the same, patients tend to choose physicians with a higher status or professional titles [18]. To a certain extent, physicians with a high-level offline status might have a heavy workload but not enough time to contribute via the internet [18]. For this reason, the log-in behaviors of physicians with high-level offline statuses have a weaker impact on patient consultation. Therefore, the following hypothesis is proposed:

Hypothesis 3: the relationship between log-in behavior and patient consultation is negatively moderated by the physician’s offline status.

When patients choose physicians on the web, they rely mostly on offline and web signals. However, most patients regard offline signals as more reliable sources and treat web signals as additional information sources that supplement offline signals. Status may have a negative moderating effect on web signals [18]. When a physician has a high status, the offline signal will be sufficient for patients to make a decision. People are willing to accept the services of physicians with a high-level status as *credence services* instead of considering the service outcome reflected in web signals [25]. As physicians with high-level statuses may attract more patients, the effect of web reviews on patient consultation will be weakened. Hence, the following hypothesis is proposed:

Hypothesis 4: the relationship between web reviews and patient consultation is negatively moderated by a physician’s offline status.

**Figure 1:**
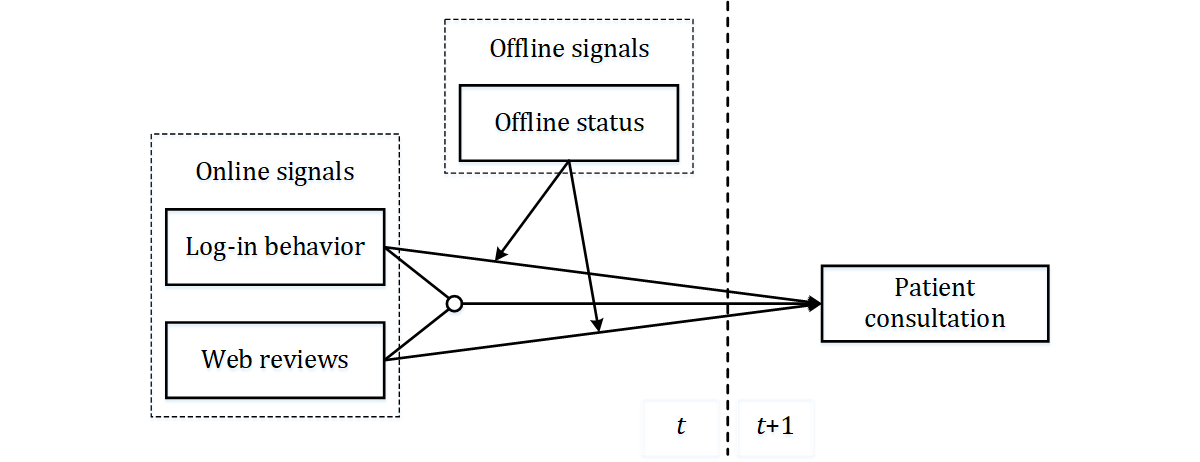
Research model.

The research model is shown in Figure 1.

## Methods

### Research Context and Data Collection

**Figure 2:**
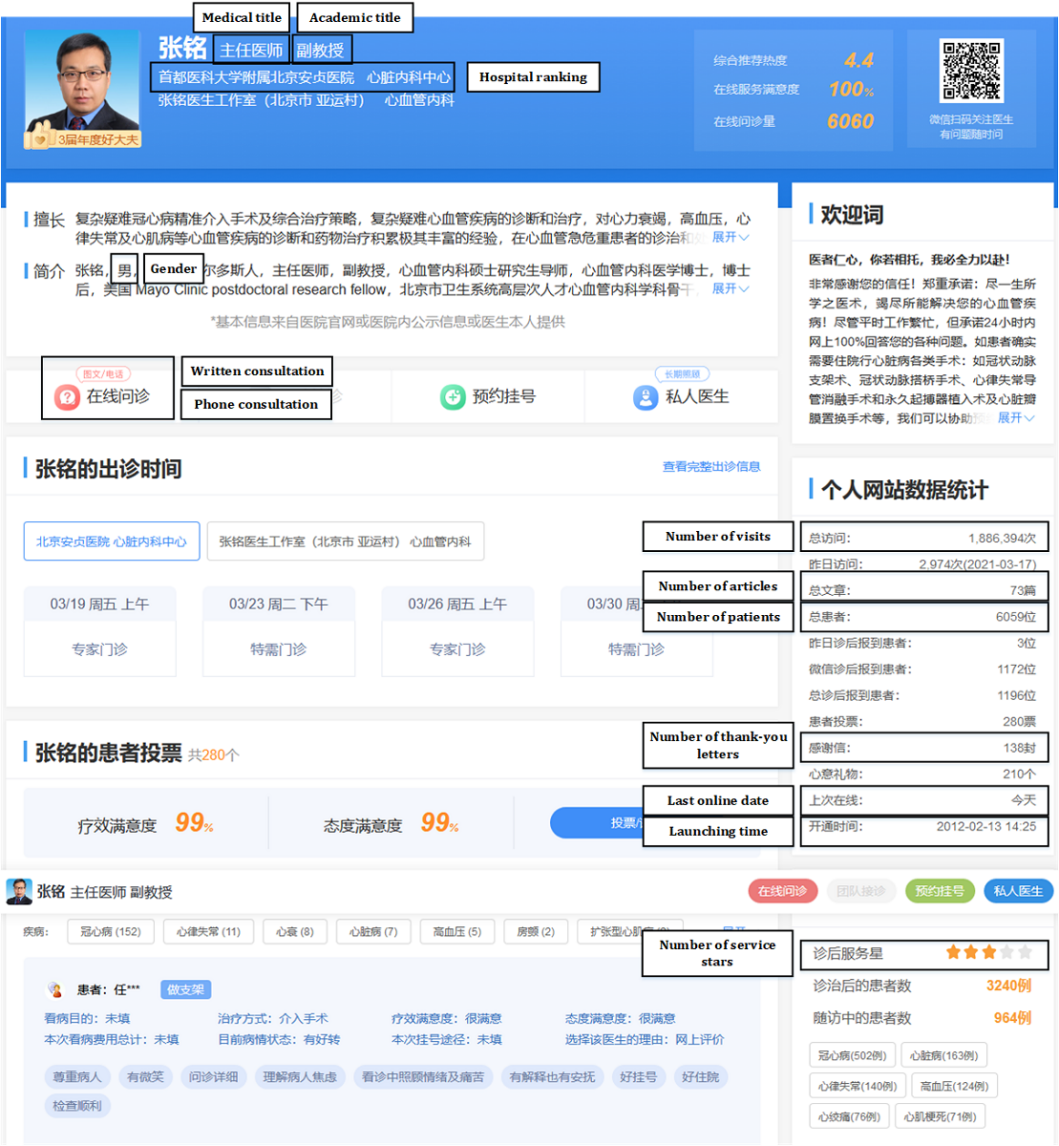
An example of physician information in online health communities.

The data used in this study were collected from Good Physician Online, which is one of the most popular and professional OHCs in China. It was founded in 2006 and, currently, more than 8000 hospitals and 500,000 physicians’ information is presented on this website. Studying such a large and popular OHC can increase the generality of the results. Moreover, physicians registered on the Good Physician Online website have a profile home page, which contains information, such as physicians’ background (name, medical title, academic title, hospital department, specialty, brief introduction, etc), patients’ reviews, and information about web-based services. The information in a physician’s profile home page is considerable and can help patients understand the physician and make a decision. Figure 2 shows an example of physician information shown on the OHC.

**Figure 3:**
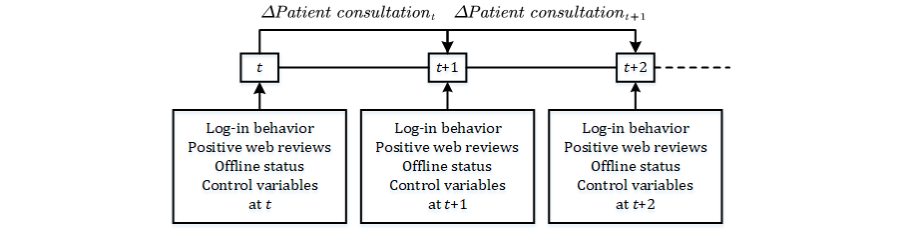
Data collection and processing.

To reduce the influence of disease types, we only included physicians who treated patients with coronary heart disease as our sample. Using web crawler technology, we collected data from February 2019 to July 2019 (once every month during these six periods), which covered public information of hospitals and physicians presented on this website. We designed a longitudinal study to investigate whether a physician’s log-in behavior and web reviews would change patient consultation choices. The data collection process is illustrated in Figure 3. After deletion of invalid data, short-panel data from 911 physicians over five periods were obtained for a total of 4555 physician data points. These physicians were currently active on the website, and the most recent log-in time was within 1 month.

### Variable Measurement

Table 2 presents the variable description. The dependent variable in this study was patient consultation. We used the number of patients before time *t* as a proxy for patient consultation, in accordance with previous research [5,14]. The number of patients included those who only consulted via the internet and those who consulted again after offline consultation. This study used the difference between the two periods as the dependent variable to reduce the causal relationship between the dependent and independent variables.

**Table 2:** Variable description.

Variables		Description	Proxy
**Dependent variable**			
	Patient consultation	Patients evaluate the information about a physician and make the decision to consult online.	Patients
**Independent variable**			
	Log-in behavior	One kind of online behavior that a physician launches his profile home page and log-ins his accounts.	Last web date
	Positive web reviews	Positive reviews written by patients who have experienced a physician's health care service.	Thank-you letters
**Moderating variable**			
	Offline status	The offline prestige of a physician in the career.	Medical title, academic title, or hospital ranking
**Control variable**			
	Gender	The gender of a physician, 0 is male, 1 is female.	Gender
	Usage years	The number of years that a physician using the OHC^a^.	Usage years
	Visits	The number of patients for visiting a physician’s profile home page.	Visits
	Articles	The number of articles that a physician post on his or her profile home page.	Articles
	Service stars	The number of service stars displayed on a physician’s profile home page.	Service stars
	Written consultation	One type of online service. If a physician provided online written service, then 1; If not provided then 0.	Written consultation
	Phone consultation	One type of online service. If a physician provided online phone service, then 1; If not provided then 0.	Phone consultation

^a^OHC: online health community.

**Figure 4:**
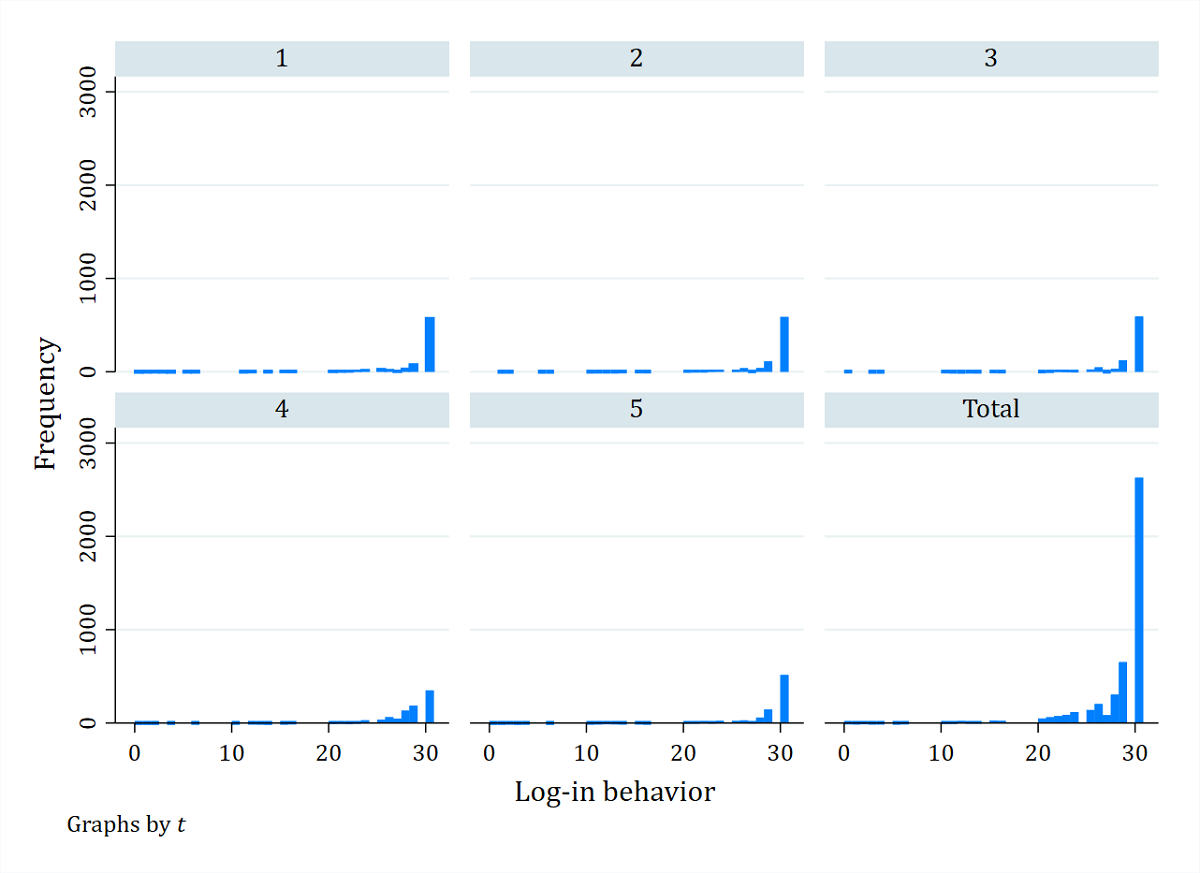
The frequency of physicians’ log-in behaviors in online health communities.

The independent variables included physician’s log-in behavior and positive web reviews. In this study, log-in behavior was measured by the last date a physician was on the web at time *t*. If the physician logged in today, the log-in behavior is marked as 30; if the last on the web date is 30 days ago, the log-in behavior is marked as 0. The values are decremented individually. Figure 4 shows the frequency statistics of physicians’ log-in behaviors over five periods. It can be seen that physicians’ log-in patterns are different, and it is worth studying.

After the web-based consultation on the Good Physician Online website, patients can express their satisfaction or dissatisfaction with the physician’s service by sharing their treatment experience and writing a thank-you letter. The difference between a treatment experience and a thank-you letter is that the latter is a positive web review, and the patient who chooses to write a thank-you letter is definitely satisfied with the physician’s service. Referring to previous research [14,27], we used the number of thank-you letters a physician received from patients at time *t* to measure the physician’s positive web reviews.

The moderating variable in our research model is offline status, which mainly reflects the offline prestige in the physician’s career. According to previous research [27,42], we used the physician’s occupational title ranking and hospital standing at time *t* as a proxy for physicians’ offline status. A physician’s occupational title indicates the duties of a physician in a hospital, which is a manifestation of the physician’s professional expertise, health knowledge, and experience. A physician’s occupational title ranking includes medical title (chief physician, deputy chief physician, attending physician, and resident physician, coded from 4 to 1) and academic title (professor, deputy professor, and lecturer, coded from 3 to 1). Hospital standing can reflect an advantage in human capital, experience, health facilities, and technology, which is ranked as 3, 2, and 1. According to the methodology of previous research [43], this study integrated three variables to represent offline status. We standardized three variables by subtracting the means and dividing by the SEs, as shown in equation (1). Thus, the offline status of a physician was measured using equation (2).

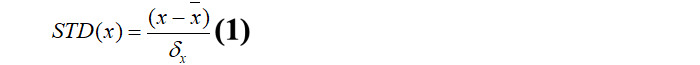



Offline status = STD [STD (medical title) + STD (academic title) + STD (hospital ranking)] **(2)**

The control variables included the physician’s gender, usage years, number of visits, articles, service stars, written consultation, and phone consultation provided before time *t*. This information about the physician has been shown to be relevant to patients making consulting choices [5,7-9,13,14]. Gender is coded with “0” for male and “1” for female. Usage years are measured by the difference between the launching time of a physician’s personal website and time *t*. The number of visits, articles, and service stars is the information displayed on the physician’s profile home page before time *t*. Written consultation and phone consultation are two important types of services that physicians can provide on the Good Physician Online website. As the distributions of visits and articles are nonnormal, *ln* (*x*+1) transformations were also used for them.

## Results

### Descriptive Statistics and Correlation Results

Tables 3 and 4 show the descriptive statistics and correlations of variables, respectively. As shown in Table 4, log-in behavior is positively correlated with patient consultation and positive web reviews, and the β coefficients were .169 and .219, respectively. Log-in behavior and web reviews have positive correlations with offline status, with β coefficients of .047 and .284, respectively.

**Table 3:** Descriptive statistics of variables (n=4555).

Variables	Values		
	Mean (SD)	Min	Max
Gender	0.157 (0.364)	0	1
Usage years	5.127 (3.176)	0	11.340
Visits	718,657.100 (1,780,896.000)	27	1.80e+07
Articles	18.704 (72.534)	0	1314
Service stars	0.818 (1.217)	0	5
Written consultation	0.503 (0.500)	0	1
Phone consultation	0.643 (0.479)	0	1
Last web-based date	28.025 (4.105)	0	30
Thank-you letters	38.225 (93.953)	0	1606
Medical title	3.095 (0.850)	0	4
Academic title	1.186 (1.241)	0	3
Hospital ranking	2.940 (0.351)	0	3
Patients	18.013 (38.372)	0	678

**Table 4:** Correlations of variables (n=4555).

Variables	Gender	Usage years	ln(Visits+1)	ln(Articles+1)	Service stars	Written consultation	Phone consultation	Log-in behavior	Reviews	Status	Consultation
Gender	1	—^a^	—	—	—	—	—	—	—	—	—
Usage years	−0.056	1	—	—	—	—	—	—	—	—	—
ln(Visits+1)	−0.068	0.717	1	—	—	—	—	—	—	—	—
ln(Articles+1)	−0.150	0.321	0.502	1	—	—	—	—	—	—	—
Service stars	−0.041	0.082	0.223	0.188	1	—	—	—	—	—	—
Written consultation	0.021	−0.118	−0.203	−0.148	−0.068	1	—	—	—	—	—
Phone consultation	0.037	−0.152	−0.268	−0.195	−0.088	0.750	1	—	—	—	—
Log-in behavior	−0.035	0.066	0.131	0.109	0.259	−0.039	−0.037	1	—	—	—
Reviews	−0.088	0.448	0.647	0.292	0.498	−0.128	−0.174	0.219	1	—	—
Status	0.148	0.443	0.388	0.117	0.097	−0.029	−0.050	0.047	0.284	1	—
Consultation	−0.032	0.125	0.288	0.210	0.562	−0.020	−0.048	0.169	0.455	0.168	1

^a^Not applicable.

### Estimation Model

As can be seen from Table 3, the dependent variables (patients) were nonnegative integers and their variance was greater than the mean; therefore, the negative binomial regression model was suitable for this study. The negative binominal probability function is as shown in equation (3), which has two parameters, θ and λ. Parameter θ captures overdispersion in the data, and parameter λ is the expected value of the distribution.

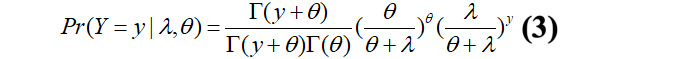



To test the hypotheses, the negative binomial regression model with fixed effects is explicitly expressed as shown in equation (4).

△*Patient consultation* = *Patient consultation_i_*_,_*_t_*_+_*_1_* − *Patient consultation_i_*_,_*_t_*

= *α*_0_ + *α*_1_*Gender_i_* + *α*_2_*Usage years_i,t_* + *α*_3_*ln*(*Visits_i,t_* + 1) + *α*_4_*ln*(*Articles_i,t_* + 1) + *α*_5_*Service stars_i,t_* + *α*_6_*Written consultation_i,t_* + *α*_7_*Phone consulatation_i,t_* + α_8_*Log-in behavior_i,t_* + *α*_9_*Positive web reviews_i,t_* + *α*_10_*Service stars_i,t_* × *Positive web reviews_i,t_* + *α*_11_*Offline status_i,t_* + *α*_12_*Log-in behavior_i,t_* × *Offline status_i,t_* + *α*_13_*Positive web reviews_i,t_* × *Offline status_i,t_* + *ε_i,t_***(4)**

Let *i*=1, 2, 3,..., *n* be the index of physicians. For equation (4), *α*_0_ to *α*_13_ are the parameters to be estimated.

### Regression Results

This study estimated the models using STATA software version 15.0 (StataCorp). The result of the Hausman test (χ^2^_14_=534.0; *P*<.001) indicated that the fixed effects model was suitable for this study. Table 5 shows the results of the fixed effects model hierarchically. Model 1 contains only constant and control variables, and model 2-model 5 add independent variables and interaction terms.

**Table 5:** Regression results (fixed effects model).

Variable	Model 1			Model 2			Model 3			Model 4			Model 5	
	α^a^ (SE)	*P* value	α (SE)		*P* value	α (SE)		*P* value	α (SE)		*P* value	α (SE)		*P* value
Constant	−1.005 (0.324)	.002	−1.449 (0.344)		<.001	−1.059 (0.474)		.02	−1.074 (0.357)		.003	−.280 (0.347)		.42
Gender	−.269 (0.098)	.006	−.266 (0.098)		.007	−.241 (0.097)		.01	−.367 (0.101)		<.001	−.338 (0.100)		.001
Usage years	.034 (0.016)	.04	.033 (0.016)		.04	.035 (0.016)		.03	.015 (0.017)		.37	.016 (0.017)		.32
ln(Visits+1)	.199 (0.031)	<.001	.199 (0.031)		<.001	.135 (0.036)		<.001	.176 (0.031)		<.001	.110 (0.036)		.002
ln(Articles+1)	−.126 (0.027)	<.001	−.126 (0.027)		<.001	−.110 (0.027)		<.001	−.118 (0.027)		<.001	−.106 (0.027)		<.001
Service stars	.118 (0.015)	<.001	.112 (0.015)		<.001	.101 (0.015)		<.001	.111 (0.015)		<.001	.106 (0.015)		<.001
Written consultation	.104 (0.086)	.001	.106 (0.031)		.001	.106 (0.031)		.001	.107 (0.031)		.001	.104 (0.030)		.001
Phone consultation	−.302 (0.086)	<.001	−.311 (0.086)		<.001	−.303 (0.085)		<.001	−.323 (0.087)		<.001	−.319 (0.085)		<.001
Log-in behavior	—^b^	—	.016 (0.004)		<.001	.014 (0.011)		.20	.016 (0.004)		<.001	—		—
Positive reviews^c^	—	—	—		—	.105 (0.122)		.39	—		—	.128 (0.037)		.001
Log-in behavior×positive reviews	—	—	—		—	.001 (0.004)		.86	—		—	—		—
Offline status	—	—	—		—	—		—	.220 (0.142)		.12	−.023 (0.088)		.80
Log-in behavior×offline status	—	—	—		—	—		—	−.001 (0.005)		.80	—		—
Positive reviews×offline status	—	—	—		—	—		—	—		—	.070 (0.027)		.009
Log likelihood	−9349.410	—	−9341.811		—	−9336.253		—	−9331.336		—	−9329.970		—
Wald chi-square (*df*)	282.6 (7)	—	296.0 (8)		—	311.9 (10)		—	317.9 (10)		—	334.1 (10)		—
*P* value	<.001	—	<.001		—	<.001		—	<.001		—	<.001		—

^a^Coefficient of the variable.

^b^Not applicable.

^c^Positive reviews: positive web reviews.

**Figure 5:**
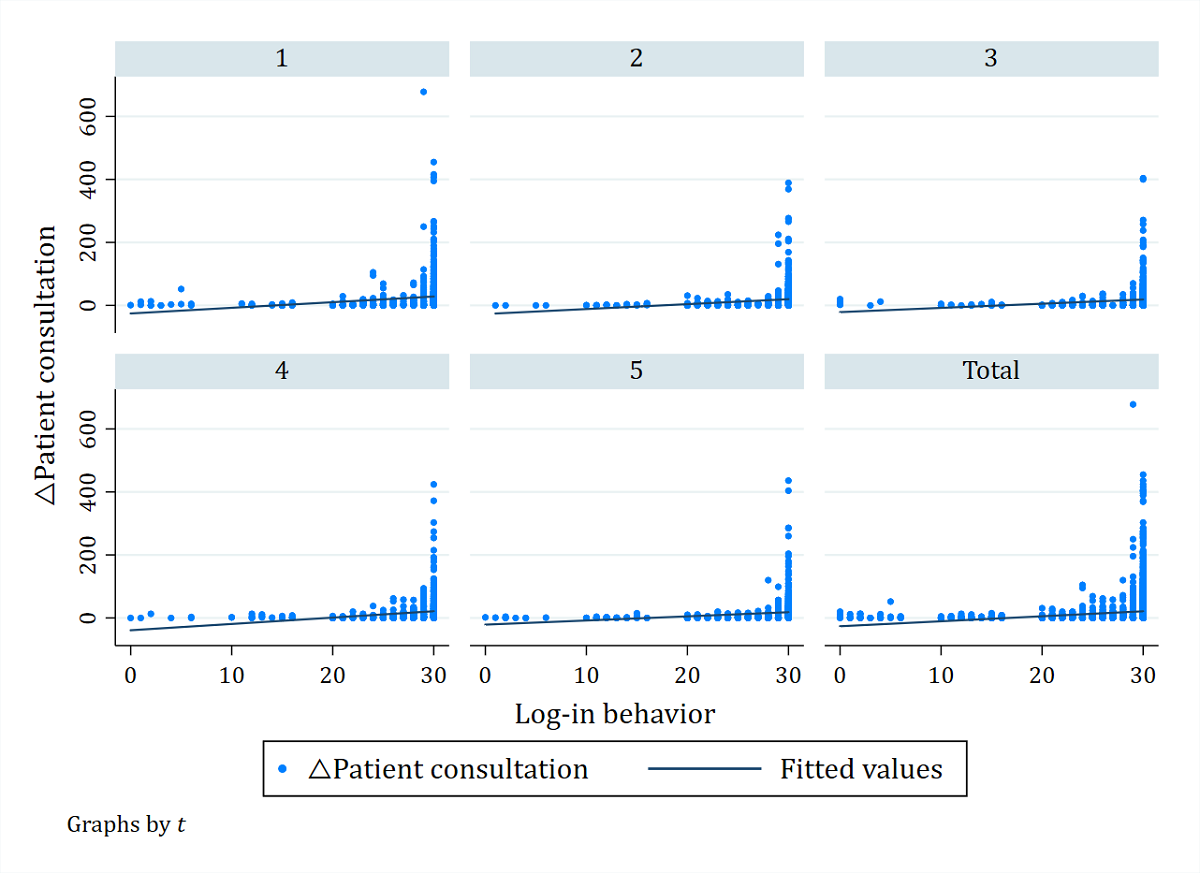
The effect of log-in behavior and patient consultation on online health communities.

From model 2, the coefficient of log-in behavior (α=.016; *P*<.001) is positive and statistically significant, which supports hypothesis 1. The effects of log-in behavior on patient consultation are shown in Figure 5. As the number of log-in behaviors increases, the number of △patient consultation increases. When log-in behavior was less than 10 or 15, △patient consultation was 0 or <0. A comprehensive view of the regression lines of the five periods shows that the value of log-in behavior is ≤10, with the lowest number of patients making consulting choices.

The results of model 3 show that the interaction between log-in behavior and web reviews (α=.001) is positive but not significant. This finding suggests that log-in behavior and web reviews do not have a complementary relationship that affects patient consultation. Therefore, hypothesis 2 is not supported.

The results of model 4 show that the interaction between log-in behavior and offline status (α=−.001) is negative but not significant. This means that the relationship between a physician’s log-in behavior and patient consultation is not negatively moderated by offline status. Therefore, hypothesis 3 is contradicted.

The results of model 5 show that the interaction between web reviews and offline status (α=.070; *P*=.009) is positive and significant. This finding means that the effect of web reviews on patient consultation is stronger for physicians with a high status. Therefore, hypothesis 4 is contradicted.

### Robustness Check

This study added the time effect to the estimation model, equation (5), and used the two-way fixed effects model to recheck the robustness of the results. Time is defined as a dummy variable, and *t*1 (February 2019) is used as the base period. The new estimation model is shown in equation (5). Table 6 shows the results of the robustness check, which are consistent with the results of the previous model (Table 5). In addition, the joint significance of the time dummy variable was tested, and it was confirmed that the time effect should be included in the estimation model. The robustness check results suggest that hypothesis 1 is supported.


△*Patient consultation* = *Patient consultation_i_*_,_*_t_*_+_*_1_* − *Patient consultation_i_*_,_*_t_*

= *β*_0_ + *β*_1_*Gender_i_* + *β*_2_*Usage years_i,t_* + *β*_3_*ln*(*Visits_i,t_* + 1) + *β*_4_*ln*(*Articles_i,t_* + 1) + *β*_5_*Service stars_i,t_* + *β*_6_*Written consultation_i,t_* + *β*_7_*Phone consulatation_i,t_* + *β*_8_*Log-in behavior_i,t_* + *β*_9_*Positive web reviews_i,t_* + *β*_10_*Service stars_i,t_* × *Positive web reviews_i,t_* + *β*_11_*Offline status_i,t_* + *β*_12_*Log-in behavior_i,t_* × *Offline status_i,t_* + *β*_13_*Positive web reviews_i,t_* × *Offline status_i,t_* + *β*_14_*t*_2_ + *β*_15_*t*_3_ + *β*_16_*t*_4_ + *β*_17_*t*_5_ + ε*_i,t_***(5)**

Let *i*=1, 2, 3,..., *n* be the index of physicians. For equation (5), *β*_0_ to *β*_17_ were the parameters to be estimated.

**Table 6:** Robustness check (fixed effects model).

Variable		Model 1			Model 2			Model 3			Model 4			Model 5	
		β^a^ (SE)	*P* value	β (SE)		*P* value	β (SE)		*P* value	β (SE)		*P* value	β (SE)		*P* value
Constant		−.896 (0.338)	.008	−1.283 (0.356)		<.001	−.736 (0.466)		.11	−.966 (0.368)		.009	.141 (0.356)		.69
Gender		−.298 (0.101)	.003	−.294 (0.100)		.003	−.236 (0.098)		.02	−.392 (0.104)		<.001	−.329 (0.101)		.001
Usage years		.067 (0.018)	<.001	.065 (0.018)		<.001	.072 (0.017)		<.001	.046 (0.018)		.01	.055 (0.018)		.002
ln(Visits+1)		.219 (0.032)	<.001	.220 (0.032)		<.001	.084 (0.038)		.02	.200 (0.033)		<.001	.062 (0.038)		.10
ln(Articles+1)		−.090 (0.029)	.002	−.090 (0.029)		.002	−.056 (0.028)		.049	−.081 (0.029)		.006	−.056 (0.029)		.049
Service stars		.133 (0.014)	<.001	.128 (0.014)		<.001	.109 (0.014)		<.001	.128 (0.014)		<.001	.112 (0.014)		<.001
Written consultation		.002 (0.040)	.96	.003 (0.040)		.94	.001 (0.039)		.98	−.001 (0.040)		.97	−.001 (0.038)		.98
Phone consultation		−.185 (0.096)	.06	−.197 (0.096)		.04	−.178 (0.094)		.06	−.202 (0.097)		.04	−.196 (0.093)		.04
Log-in behavior		N/A^b^	N/A	.014 (0.004)		.001	.020 (0.010)		.06	.015 (0.004)		<.001	N/A		N/A
Positive reviews^c^		N/A	N/A	N/A		N/A	.346 (0.114)		.002	N/A		N/A	.278 (0.040)		<.001
Log-in behavior×positive reviews		N/A	N/A	N/A		N/A	−.003 (0.004)		.46	N/A		N/A	N/A		N/A
Offline status		N/A	N/A	N/A		N/A	N/A		N/A	.294 (0.135)		.03	−.141 (0.092)		.13
Log-in behavior×offline status		N/A	N/A	N/A		N/A	N/A		N/A	−.004 (0.004)		.36	N/A		N/A
Positive reviews×offline status		N/A	N/A	N/A		N/A	N/A		N/A	N/A		N/A	.109 (0.028)		<.001
**T**															
	2	−.391 (0.024)	<.001	−.391 (0.024)		<.001	−.389 (0.024)		<.001	−.392 (0.024)		<.001	−.390 (0.023)		<.001
	3	−.443 (0.033)	<.001	−.442 (0.033)		<.001	−.452 (0.032)		<.001	−.444 (0.033)		<.001	−.451 (0.031)		<.001
	4	−.461 (0.025)	<.001	−.456 (0.025)		<.001	−.472 (0.024)		<.001	−.451 (0.025)		<.001	−.467 (0.024)		<.001
	5	−.540 (0.025)	<.001	−.537 (0.025)		<.001	−.560 (0.025)		<.001	−.530 (0.025)		<.001	−0.552 (0.024)		<.001
Log likelihood		−9103.221	N/A	−9096.944		N/A	−9075.380		N/A	−9087.928		N/A	−9064.380		N/A
Wald chi-square (*df*)		1001.0 (11)	N/A	1015.4 (12)		N/A	1103.6 (14)		N/A	1040.8 (14)		N/A	1161.3 (14)		N/A
*P* value		<.001	N/A	<.001		N/A	<.001		N/A	<.001		N/A	<.001		N/A

^a^Coefficient of the variable.

^b^N/A: not applicable.

^c^Positive reviews: positive web reviews.

## Discussion

### Principal Findings

In contrast to previous studies on physicians’ web-based behaviors, our research focused on log-in behavior and found that it had a positive effect on patient consultation. The results were consistent with those of Li et al [31], who believed that physicians with higher-frequency log-ins are more likely to attract patients, because they seem to be more responsible and have a timely service process. The results also indicated that physicians’ web-based behaviors positively influence patients’ consulting choices [6,9,13], including log-in behavior. Our research also found that when a physician did not log in to the OHC for more than 20 days, the number of patients who chose them was small, even 0.

Our research used web reviews generated by patients after receiving health care services as a web signal to represent service outcomes. Our research found that a physician’s log-in behavior and web reviews did not have a complementary relationship in affecting patient consultation, which was different from the findings of previous research on service quality [12,14]. On the one hand, it may be that log-in behavior and web reviews have separate effects on patient consultation, and patients do not consider both. On the other hand, although log-in behavior is a web-based behavior, it may not be directly related to the delivery process of a physician’s response to consultation.

Patients mostly rely on both offline and web signals to choose a physician. This study found that web reviews were positively moderated by offline status. This is inconsistent with the findings of previous research, which suggests that web signals should be negatively moderated by offline signals [18]. However, offline status cannot moderate log-in behavior. A possible explanation is that most patients view offline signals as a more reliable source than web signals. Compared with the degree of initiative and effort, offline prestige (ie, offline status) in a physician’s career can better reflect the service outcome quality.

### Theoretical Implications

This study offers theoretical contributions in the following ways. First, previous studies have explored the influencing factors related to patients’ consultation choices, including some web-based behaviors of physicians, such as publishing articles, providing written consultation, and phone consultation. However, the literature on the role of physicians’ log-in behavior is inadequate. Logging is the central working sphere and is the first step for a physician to provide health care services. Log-in behavior represents the central effort, activeness, and service process quality. Our research found that log-in behavior could influence patients’ consulting choices. This finding extends the understanding of physicians’ web-based behaviors and may also be used in other service fields.

Second, although some signaling literature in the context of eHealth has discussed web reviews, no research considers web reviews as service outcomes with the log-in behavior of physicians. However, this study found that log-in behavior and web reviews did not have a complementary relationship that affected patient consultation. Therefore, these findings contribute to research on patient consultation in OHCs.

Third, a clear distinction exists between web and offline signals. This study investigated the main effects of web signals (log-in behavior and web reviews) and their interactions with offline signals (offline status). The results revealed that the moderating effects of offline status on these two signals were different. From this perspective, this study extended the understanding of multiple signal interactions.

### Implications for Practice

This study has several practical implications. First, for health care service providers, our evidence-based research demonstrates that log-in behavior is also an important factor in influencing patients’ choice of consultation. Apart from other web-based behaviors, patients can judge a physician’s activeness, efforts, and service process quality by relying on their log-in behavior. Physicians should value their web-based behaviors and log in to OHCs proactively, transmitting signals of active participation and timely responses to patients. Furthermore, operators of OHCs should pay attention to physicians’ log-in issues. The more actively physicians participate in web-based platforms, the more successful the OHCs will be.

Second, the results show that log-in behavior and web reviews do not have a complementary relationship that affects patient consultation. Physicians should distinguish between log-in behavior and other web-based behaviors. Although web-based behaviors can reflect a physician’s activeness and effort, there may be differences in service process quality.

Third, the results show that multiple signals from different signaling mechanisms affect patient consultation. Offline signals can have positive moderating effects on web signals. Hence, physicians should value the impacts of both web-based and offline service quality, and offline service quality is more credible than web-based service quality for patients.

### Limitations and Future Research

This study has certain limitations. First, this study used physician data from only one OHC and one disease type. However, interpretation of the results may be limited. Therefore, it is necessary to collect data from physicians with various expertise on different platforms simultaneously to further verify the research model. Second, the study used the physician’s last date displayed on the web on the day of crawling data to measure log-in behavior, which has certain limitations. In future research, we could measure log-in behavior through other methods, such as counting physicians’ log-in times within a month. Third, the control variables selected in this study may have ignored some important variables, especially those related to patients. As websites tend to obscure customer names to protect privacy, it is difficult to obtain these data from the website.

### Conclusions

Drawing on the signaling theory, this study explores the effects of physicians’ log-in behavior and web reviews on patient consultation in OHCs. This study hypothesized that two signals (ie, log-in behavior and web reviews) and their interaction affect patients’ consultation choices, and the relationships between web signals and patient consultation were moderated by offline signals (ie, offline status). Short-panel data over five periods were used to test these hypotheses. Our research found that a physician’s log-in behavior positively affects patient consultation, and a physician’s no–log-in days should be no more than 20 days. Log-in behavior and web reviews had no complementary relationship that affects patient consultation. Furthermore, offline status could only positively moderate web reviews instead of log-in behavior.

## Abbreviations

OHC: online health community
